# How do drivers mitigate the effects of naturalistic visual complexity? On attentional strategies and their implications under a change blindness protocol

**DOI:** 10.1186/s41235-023-00501-1

**Published:** 2023-08-09

**Authors:** Vasiliki Kondyli, Mehul Bhatt, Daniel Levin, Jakob Suchan

**Affiliations:** 1 CoDesign Lab EU – codesign-lab.org, Örebro University, Örebro, Sweden; 2 Vanderbilt University, Nashville, USA; 3 German Aerospace Center - DLR, Institute of Systems Engineering for Future Mobility, Oldenburg, Germany

**Keywords:** Visual perception, Change blindness, Visuospatial complexity, Attentional strategies, Naturalistic observation, Everyday driving

## Abstract

How do the limits of high-level visual processing affect human performance in naturalistic, dynamic settings of (multimodal) interaction where observers can draw on experience to strategically adapt attention to familiar forms of complexity? In this backdrop, we investigate change detection in a driving context to study attentional allocation aimed at overcoming environmental complexity and temporal load. Results indicate that visuospatial complexity substantially increases change blindness but also that participants effectively respond to this load by increasing their focus on safety-relevant events, by adjusting their driving, and by avoiding non-productive forms of attentional elaboration, thereby also controlling “looked-but-failed-to-see” errors. Furthermore, analyses of gaze patterns reveal that drivers occasionally, but effectively, limit attentional monitoring and lingering for irrelevant changes. Overall, the experimental outcomes reveal how drivers exhibit effective attentional compensation in highly complex situations. Our findings uncover implications for driving education and development of driving skill-testing methods, as well as for human-factors guided development of AI-based driving assistance systems.

## Significance

Previous research has demonstrated that people frequently fail to detect changes as they interact with the environment and with other people. However, in many cases people manage to maintain situation awareness and effectively cope with complex everyday activities such as driving in visually complex environments. In this work we use an embodied simulated driving task to document how drivers can adapt their visual attention by selectively focusing on safety-critical events over less critical events. We suggest that this knowledge can support the development of attentional diagnostics for effective driving instruction and that it is crucial for the development of human-centred technologies and autonomous systems that are capable of anticipating the behaviour of drivers and other road users.

## Introduction

Visuospatial attention is critical in many everyday activities, especially those involving embodied multimodal interactions, both between humans and with the surrounding environment. One of the most important of these activities is driving which includes a number of required tasks, such as maintaining visual awareness of the surrounding environment, planning the driving trajectory, and executing control actions such as steering and braking. These and other tasks impose a range of visual-cognitive processing demands on the driver (Underwood, 2007; Vallières et al., 2015). For example, drivers tend to steer in the direction of their gaze (Robertshaw & Wilkie, 2008) and fixations are clustered near the focus of expansion when driving in a straight trajectory (Mourant & Rockwell, 1970). These perceptual challenges are combined with complex higher-level tasks such as active search guided by the semantic content of the environment (e.g. intersections, signs, traffic lights, Shinoda et al. (2001), Findlay and Gilchrist (2003)). In situations where continuous visual awareness is critical, these attentional demands can exceed the available resources, causing performance to deteriorate, limiting the effectiveness of visual search for potential hazards (Norman & Bobrow, 1975; Wickens, 2008; Brookhuis & de Waard, 2010; Fuller, 2005; Palmiero et al., 2019).

In this paper, we assess the attentional demands on drivers using a change detection task (Simons & Levin, 1998). Performance in attentionally demanding tasks such as change detection is affected by several internal and external factors. Internal factors pertain to age, physical visual and cognitive limitations, e.g. visual neglect, stroke, cognitive decline (Ball et al., 1993; Plummer et al., 2020), task-related experience (Beck et al., 2012; Maturi & Sheridan, 2020), and familiarity (Charlton & Starkey, 2013). A number of external factors also affect visual processing and ultimately the task performance. Depending on the type of stimuli investigated (e.g. static or dynamic, 2D or 3D, visual or auditory), external factors comprise specific properties of the targets or the background, such as size, or clutter (Beck et al., 2010; Park et al., 2015; Beanland et al., 2017). In the experiment reported here, we asked participants to detect visual changes during a naturalistic simulated driving task. There were two main objectives: first, to assess the degree to which participants effectively controlled their attention in response to external factors including environmental complexity and the temporal relationships between changes; and second, to analyse participants’ eye movements in order to reveal the attentional strategies they used to meet these demands.

### Visuospatial complexity in the driving scene

We refer to the variability and abundance of visual and spatial information encountered in dynamic three-dimensional environments as visuospatial complexity. Visuospatial complexity is an extension of visual complexity and it has been conceptualised using different levels of analysis ranging from pixel and shapes, to visual semantics of naturalistic images, and, most recently, by assessing three-dimensional natural and virtual environments (Foulsham et al., 2014; Li et al., 2016; Mital et al., 2011; Prpic et al., 2019; Kristjánsson, 2015). A range of metrics and tools have been developed to compute specific aspects of visuospatial complexity. These commonly rely on analyses of clutter (Moacdieh & Sarter, 2015; Rosenholtz et al., 2007), edge density (Machado et al., 2015), and symmetry (Suchan et al., 2016, 2018) among other variables, and they have been validated by assessing how effectively they predict human perceived complexity (Heaps & Handel, 1999; Da Silva et al., 2011).

The effect of visual complexity on human performance has been studied from various perspectives, including cognitive science (Harper et al., 2009), marketing (Pieters et al., 2010), psychology (Heaps & Handel, 1999; Cassarino & Setti, 2016), human-computer interaction (Tuch et al., 2009), and aesthetics (Braun et al., 2013). By most accounts, visuospatial complexity interferes with task performance. Many studies have demonstrated that a quantitative increase in basic perceptual aspects of visuospatial complexity negatively influences detection and search performance. For instance, increased visual clutter measured by the number of elements and crowding, leads to slower, less accurate visual search performance (Rosenholtz et al., 2007; Beck et al., 2010). Structural aspects of the scene such as the spatial layout of features (Beck & Trafton, 2007), and the shape of the virtual crowd, object occlusion and background (Bravo & Farid, 2004; Wolfe et al., 2002), also affect visual attention. Moreover, dynamic aspects of the environment, such as the trajectory of search target (Matsuno & Tomonaga, 2006; Rosenholtz et al., 2007), or the number of targets people need to track in time and space have been shown to negatively affect performance (Pylyshyn & Storm, 1988). Eye-tracking studies have shown that gaze behaviour can reveal the impact of environmental complexity on attentional patterns (Henderson et al., 2009; Ognjanovic et al., 2019; Perez & Bertola, 2011). For instance, an increase in clutter is correlated with an increase in fixation’s duration and the number of fixations (Beck et al., 2010, 2012).

Although a number of studies suggest that visuospatial complexity impairs perception, there are cases where complexity might also improve perception (Ellis & Turk-Browne, 2019). This is particularly true in cases where visuospatial complexity can be organised by higher-level scene semantics (Walshe & Nuthmann, 2014; Wang et al., 2010). For instance, in the context of architecture design, complexity can be a metric of richness and stimulation, and it is also related to more coherent and easier-to-navigate environments (Kaplan et al., 1989). This is because complexity provides scaffolding or structure (e.g. schematic or hierarchical structures) that constrains and supports sensory processing. Perceptual load theory suggests that complex stimuli (e.g. more distractors, greater similarity between targets and distractors) drain surplus perceptual resources and thus reduce task-irrelevant processing (Lavie & De Fockert, 2003). However, in this case, complexity does not necessarily improve overall performance but limits the effect of distractors on perceptual load (Murphy et al., 2016). It is, therefore, possible that visuospatial complexity is related to perceptual function via an inverted-U-function, suggesting that moderate levels of complexity can serve to ground and facilitate perception and memory (Kidd et al., 2012; Ellis & Turk-Browne, 2019).

### Perceptual limitations and strategies

Perceptual limitations are crucial for everyday tasks, such as driving, where change detection and visual search performance are tightly connected to traffic safety. Domain-specific investigations such as in traffic safety vis-a-vis driver’s (in)attention have explored phenomena linked to limitations of visual processing such as “attentive blank stares” or “looked-but-failed-to-see” (LBFTS) errors denoting failures to notice changes in a visual scene despite looking at the area of change (Hills, 1980; Caplovitz et al., 2008; Wolfe, 2021; Fudali-Czyc et al., 2014). Even though visuospatial complexity is one of the external aspects that can limit performance, humans can often compensate for these limitations. This may explain why ambient environmental complexity or a secondary task sometimes causes minimal disruption of a primary task such as driving (Stinchcombe & Gagnon, 2010; McCarley et al., 2004). Empirical studies examining how humans solve apparent resource conflicts suggest that people can, in many cases, compensate for their perceptual limitations either by changing attentional strategies or by prioritising attention based on the significance of the task (Jovancevic-Misic, 2008). For example, pedestrians may compensate for a secondary task during walking by reducing gait speed (Yogev-Seligmann et al., 2010). Similarly, attentional overload during driving can be compensated for slowing down the car to avoid increased accident risk (Brookhuis & de Waard, 2010; Fuller, 2005; Palmiero et al., 2019). Consequently, altering cognitive engagement and allocating more attention to difficult and risky tasks (Janssen & Brumby, 2015) indicates human abilities for strategic attentional compensation and emphasises the use of attention for dealing with complexity with selective allocating processing resources (Kimura et al., 2022). Extensive literature on stimuli relevance specifically for the case of driving behaviour suggests that hazards receive more attention than other street objects such as street signs, and that attention is easier distracted away from these objects rather than hazardous events (Garrison, 2011; Regan et al., 2013). However, the interaction between visuospatial aspects and areas of relevance during driving is not sufficiently explored.

As external complexity can positively or negatively affect human perception and trigger different cognitive mechanisms, investigating complexity requires setting the appropriate context with respect to environmental and task constraints. In this research, we consider the case of driving in urban environments as an example context for empirical investigation. A driver’s ability to quickly detect important targets, such as traffic signs, road markings, and pedestrian crossings, while ignoring irrelevant distractors (e.g. advertisements) is a key component for safe driving (Borowsky et al., 2008). Consider the case of a driver navigating a busy street, and a pedestrian on the sidewalk talking on the phone, when suddenly the pedestrian steps onto the street. Detecting the change in the behaviour of the pedestrian is crucial for the driver in this scenario. However, the driver may fail to detect the change if multiple people are walking close to the street, or if the driver is monitoring a motorcyclist overtaking the car at the same timeframe. Change detection tasks are an embedded part of everyday driving, and they require successful allocation of attention in specific areas of interest (AOIs) (Simons, 2000; Richard et al., 2002), or in some cases monitoring attention in these areas (Lochner & Trick, 2014; Pylyshyn & Storm, 1988).

**Fig. 1:**
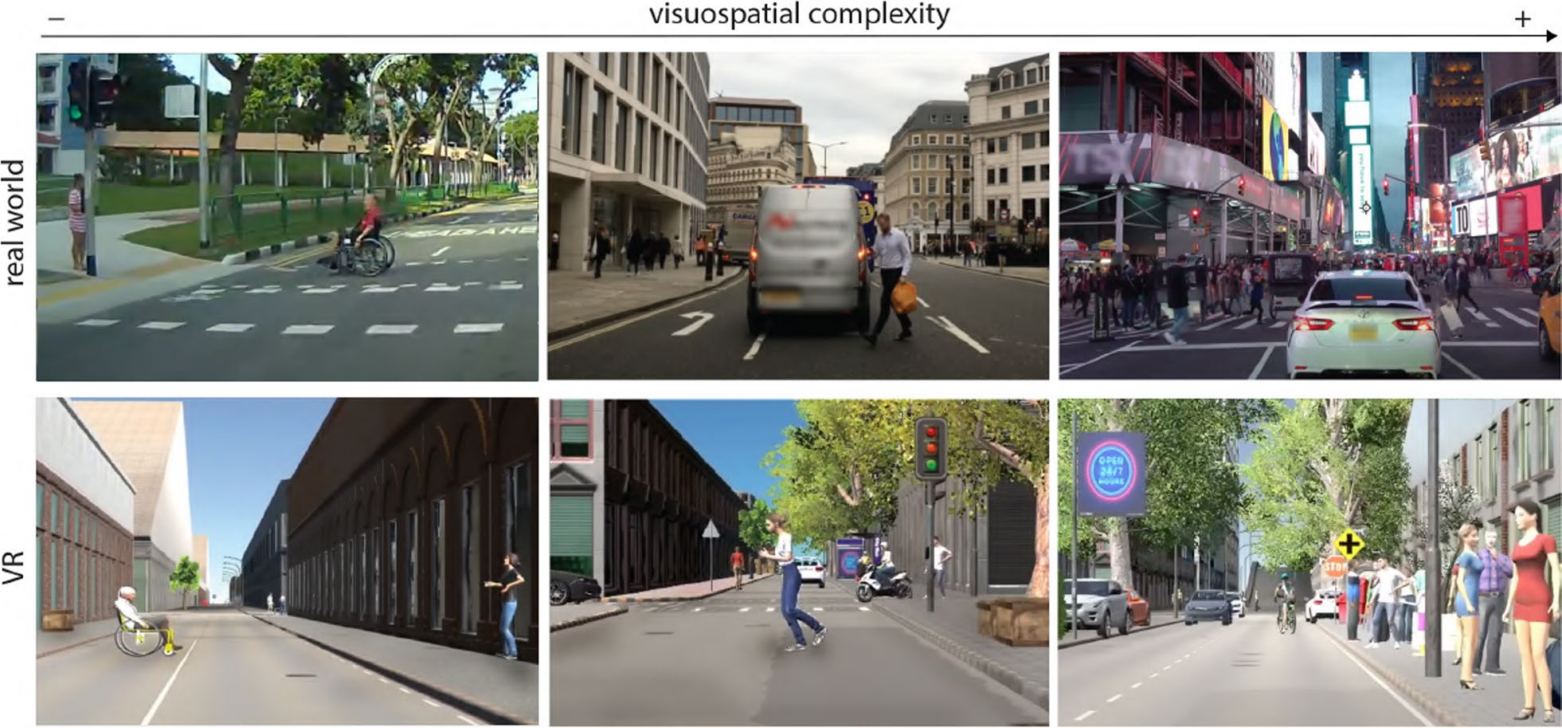
The variety of environments and incidents developed in the study are based on our systematic analysis of real-world dynamic scenes from around the world (Kondyli et al., 2020; Kondyli and Bhatt, 2020). The analysis and modelling of the multimodal interactions and the visuospatial complexity of the streetscape was the basis for the replicated scenes in VR

Failure in change detection is more likely to arise when attention is diverted or overloaded (Hyman et al., 2010). Errors can also occur due to the repetitive nature of driving, when the environmental circumstances allow it (for example in environments with very low visuospatial complexity), making drivers more susceptible to errors caused by inattention and distractions (Duncan et al., 1991; Shinar et al., 1998; Wickens, 2002). A number of studies on drivers’ behaviour examined additional aspects of external complexity that add to the workload, such as visuospatial complexity aspects in combination with the congruency of the targets, or specific events occurring in the streetscape (e.g. crossing an intersection, overtaking). The behaviour analysis in these circumstances showed that gaze and driving performance can effectively adjust to the needs of the task in many occasions (Pammer & Blink, 2013; Stinchcombe & Gagnon, 2010; Ericson et al., 2017) (Fig. 1).

### Temporal proximity and event perception

Failure in detecting changes in the surrounding environment can also be attributed to temporal proximity between events. A growing body of research suggests that fine-grained event perception can be insensitive to brief temporal disturbances, meaning that events occurring with temporal delays of milliseconds up to a few seconds might be treated by many parts of the visual-cognitive systems as equivalent and so rapid succession of events leads to an almost universal degradation of detection performance. Specifically, according to research on dual-task interference in sensory consolidation and response selection (e.g. the psychological refractory period, Pashler (1994); Raymond et al. (1992)), when two targets are presented in a time window of less than 100 ms, humans fail to encode the stimuli as two separate events (Shallice, 1964; VanRullen & Koch, 2003). Similarly, at a temporal proximity of 100–500 ms, observers failed to report which stimulus was the first or second to appear, an effect known as the attentional blink (Sheppard et al., 2002; Raymond et al., 1992). While this work focuses on short perceptual integration windows, research on event perception assess the impact of disturbances in larger event-integration windows that might extend for several seconds. According to event segmentation theory (Zacks et al., 2007) and the longstanding idea of a “psychological present”, temporal sequence between short events in a several-seconds window may be represented by default and can be immediately perceived (James, 1982). As confirmed by Pöppel (2009) and Fairhall et al. (2014), conscious activities are integrated within 2–3 s windows, however, the task is getting more difficult in longer time windows. The effect of time proximity on event perception along the time window of a few seconds has not been thoroughly tested, leaving open questions on event perception, working memory capabilities, and the role of attentional blink as a cognitive strategy rather than a resource limitation (Wyble et al., 2009).

### The present study

**Fig. 2:**
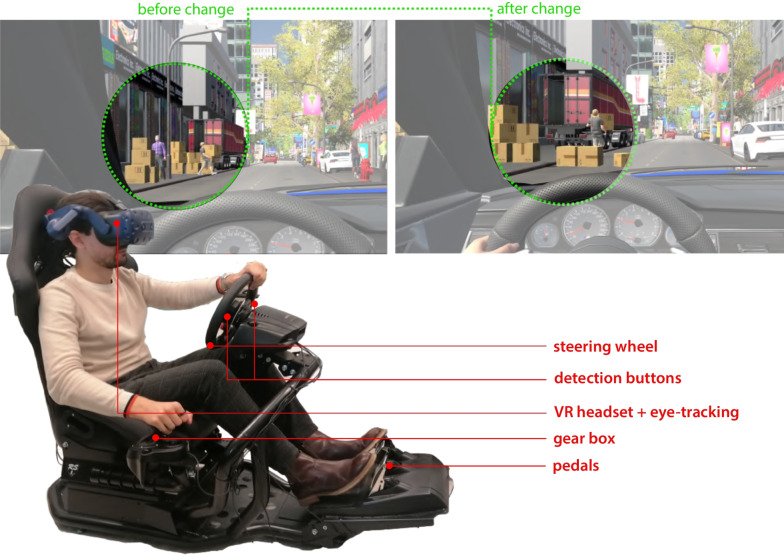
The driving simulator is equipped with VR headset and eye-tracking. The screenshots from the VR environment show an example of a behaviour change

Although visuospatial complexity of the environment can interfere with visual processing tasks, it remains unclear under which circumstances precisely (e.g. pertaining to the nature of the visual target, task difficulty, temporal load) humans do exhibit their limitations in visual processing performance, and how do they overcome the effects of visuospatial complexity during an attention regulation task. Using the change detection paradigm (Simons & Levin, 1998; Martens, 2011), this research examines the effects of visuospatial complexity in a change detection task designed and embedded within a naturalistic everyday driving experience (implemented in virtual reality). Considering the nature of the everyday driving experience, we take the diversity of typical driving events into consideration and provide a number of interactive scenarios in the change detection task (Fig. 2). The empirical study is developed to investigate two main hypotheses (Hypotheses A–B):

### Hypothesis A: Compensation strategies

We hypothesise that visuospatial complexity negatively affects the change detection performance and alters the driving behaviour. We expect that the effect of visuospatial complexity will also depend on the relevance of the change to the driving task. We systematically manipulate the types of changes and the levels of visuospatial complexity (based on a cognitive model, Kondyli et al. (2020)). We investigate the interaction between these two variables and we further explore how people adjust their performance accordingly.

### Hypothesis B: Attentional engagement and temporal proximity

We hypothesise that visuospatial complexity and temporal proximity between changes lead to adjustments in gaze behaviour along the course of events. We investigate how the gaze behaviour (e.g. fixations, gaze on AOIs, LBFTS errors) adjusts in different conditions and if people develop anticipatory or monitoring attention for the different types of changes. We manipulate the types of changes involving different agents (e.g. pedestrians, cyclists, kids, teenagers, older adults, people in wheelchairs) and street objects (e.g. parked cars, bus stops, trees). We also systematically manipulate the time proximity between the changes to test the effect of time proximity on change detection performance. We expect that a shorter time gap between changes results in worse detection performance for the second change and that the change type guides this attentional engagement.

## Method

**Fig. 3:**
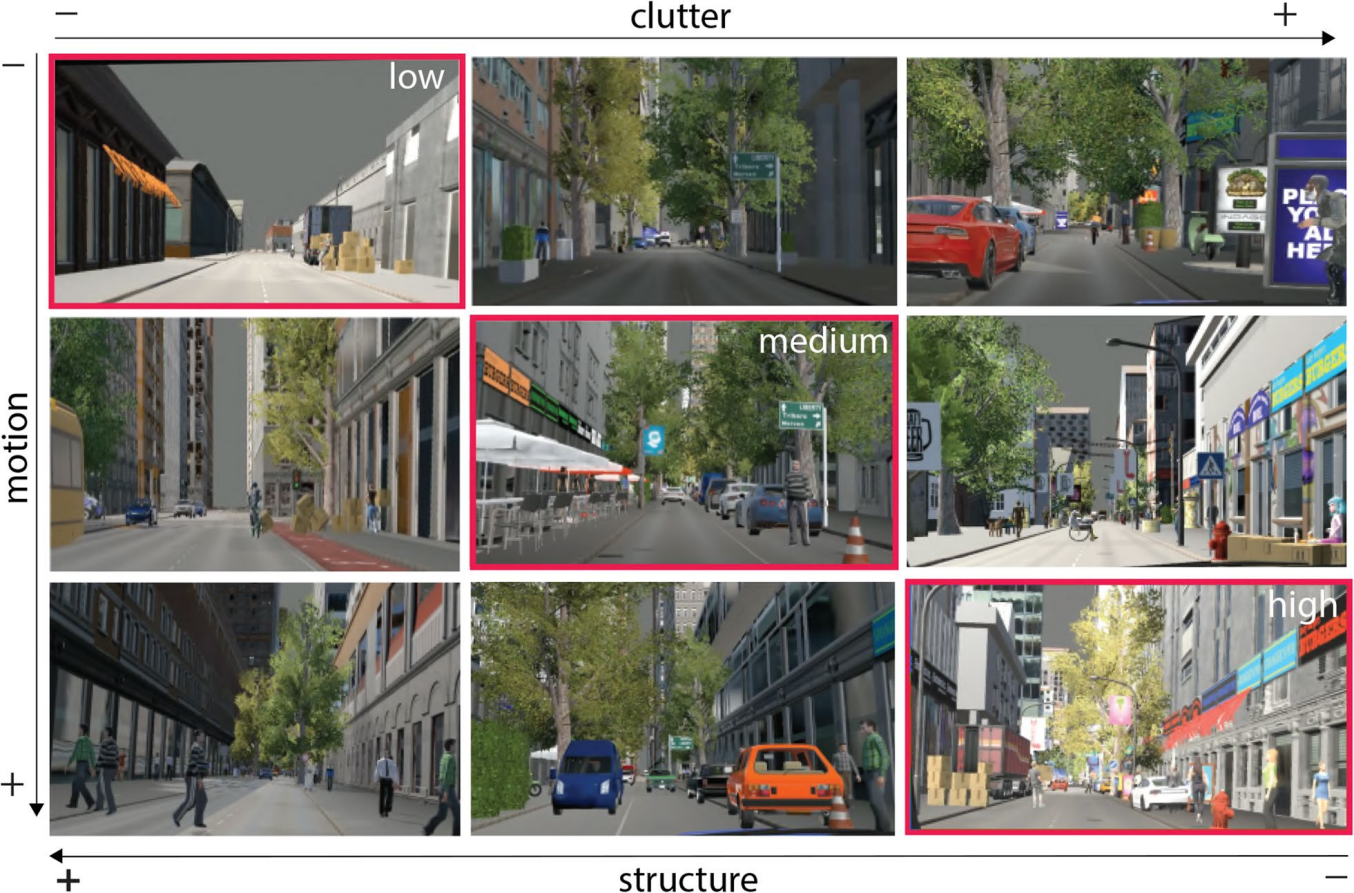
A matrix of driving environments illustrating the range of scenes created based on the visuospatial complexity model viewed from the driver’s perspective. The three chosen levels of complexity used in the developed VR environment are annotated with a red rectangular. The relationship between each aspect with the overall complexity level has been examined in previous work, referred to as the visuospatial complexity model

We develop a naturalistic experiment implemented within fully immersive virtual reality (VR) consisting of simulated driving together with immersive eye-tracking and other data collections (Fig. 3; and Table 1). Within the VR experiment, we systematically employ three variables capturing the visuospatial and interactional complexity of everyday real-world driving situations:

The first variable concerns the levels of environmental complexity defined based on a combination of visual and spatial characteristics, and guided by a cognitive model of visuospatial complexity (details in “Appendix A”). The second variable concerns the type of changes that participants were asked to detect during the driving experience, classified as behaviour-relevant, behaviour-irrelevant, or property change. The third variable concerns the temporal proximity between the changes ranging from 0 to 8 s.

We combine detection performance analysis, as measured via button presses on the steering wheel (Fig. 3), with gaze behaviour analysis (as measured via eye-tracking) to investigate the attentional strategies employed by participants towards mitigating the impacts of visuospatial complexity. A summary of the variables and metrics employed is included in Table 1.

### Participants

85 participants completed the simulation driving study; data from five participants was excluded from the analysis for technical issues, incompleteness, etc. Therefore, the analysis involves 80 participants (59 male, 21 female), between 17- and 45-year-old ($M = 25$, SD = 6.25) members of the local community or university students who voluntarily participated in the study (and were unfamiliar with the specific context of this study, or even with behavioural research in perception in general). All participants had normal or corrected-to-normal vision. 87% of the participants were licensed drivers. 47% of the participants were experienced frequent drivers (driving every day, or at least a few days per week), 38% driving a few times per month, and the rest 15% did not drive regularly (a few times per year or less).

**Table 1:**
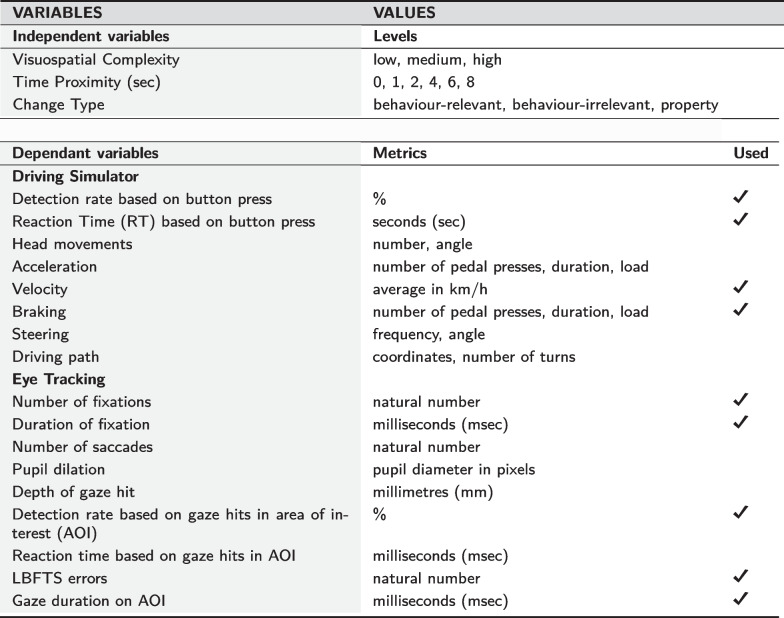
Parameters recorded for multimodal behaviour analysis and the selected ones used for the current statistical analysis. Further analysis of driving behaviour will be reported separately, and it is considered out of the scope of this publication

### Stimulus and task

While performing the simulated driving task, participants successively encountered three levels of visuospatial complexity within the virtual environment, characterised as low, medium, and high complexity environments (Fig. 3). The definition of the levels was based on the previously defined visuospatial complexity model, presented in Kondyli et al. (2020, 2021), that incorporates visual and spatial aspects of the dynamic driving environment such as the size of the street, clutter, motion, structural characteristics as well as auditory cues (details in “Appendix A”). The participants were organised into three groups based on the order in which they encountered the environments in their path: Group A (medium–high–low), Group B (high–low–medium), and Group C (low–medium–high) (Appendix-Fig. 12). To mitigate learning effects, we designed iterations of the changes occurring in the three complexity levels with slight differences in the interactions of the virtual agents, the objects, and other characteristics of the objects. The different iterations of the events also involved balancing between the right and left side of the street, female and male agents, variation of colours in clothing, etc. (details are included in Appendix-Tables 4, 5, 6).

The change detection task is organised vis-a-vis the types of changes encountered as follows: participants encountered 72 changes distributed along the driving route; of these, 36 changes were *behaviour changes*, and the rest were *property changes* (Appendix-Table 3). *Behaviour changes* pertain to sudden changes in the behaviour of a street user, whereas *Property changes* are defined as changes in the properties of an object located in the surrounding environment, essentially as sudden unrealistic changes of the environment that do not interfere with the driving task (e.g. a tree on the sidewalk changed size, or a parked car changed colour).^1^

**Fig. 4:**
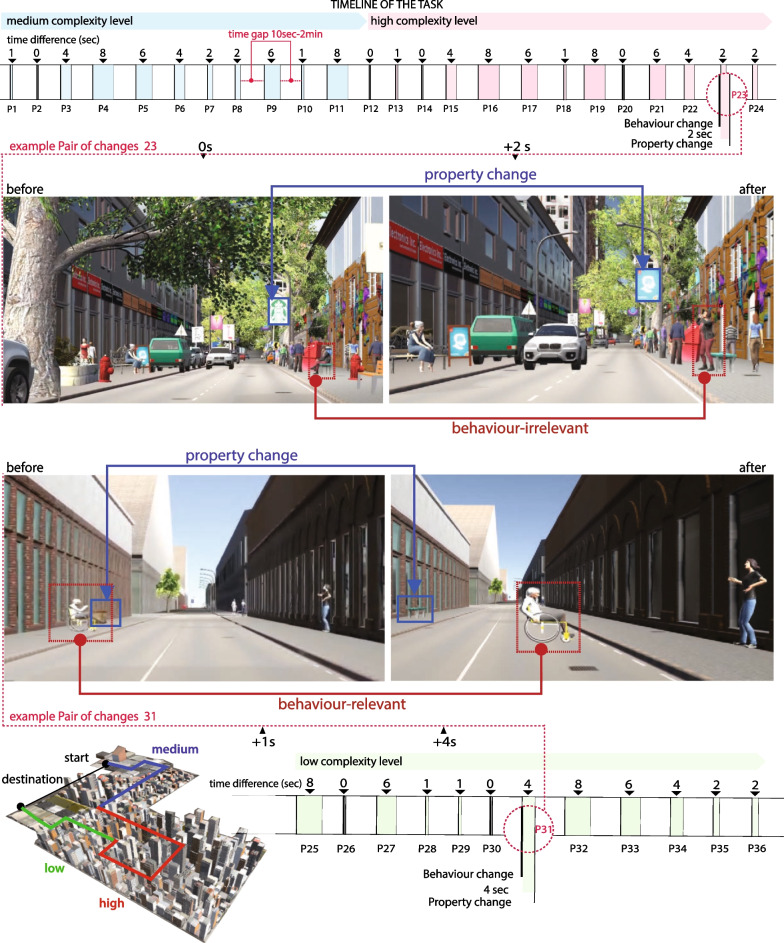
Structure of the experimental session: The horizontal line represents the timeline of the experiment for Group A (details on the order of the changes for all groups presented in Appendix, Fig. 12). Each vertical line represents one change occurring at a designated time point along the timeline. Two pairs of changes are illustrated in detail before and after the change occurs, including female agents for the behaviour changes and statics objects for the property changes

Behaviour changes are further categorised into *behaviour-relevant changes* and *behaviour-irrelevant changes*. ***Behaviour-relevant changes*** are defined as changes in the behaviour of a road user in a manner that interferes with the diving task and may involve overt latent hazards (e.g. a pedestrian walks on the sidewalk along the participant’s car when suddenly changes direction and walks towards the street). ***Behaviour-irrelevant changes*** are defined as changes in which a road user (e.g. pedestrians, cyclists) changes behaviour in a manner that does not interfere with the core driving task (e.g. a pedestrian walks on the sidewalk parallel to the participant’s car and then falls to the ground). The two groups of changes are designed to not differ significantly before the change occurs. In both groups of changes the agents perform similar activities (siting, talking, standing) before the change, they are positioned close to the road, and they are facing different directions (e.g. towards the road, opposite to the road, towards another agent, Fig. 4). The selection of behaviour changes was based on the analysis of real-world scenarios (Kondyli & Bhatt, 2020) and safety-critical situations extracted from safety reports of the German and the European transportation council’s assessment for failures in interactions between the different road users (e.g. pedestrians, cyclist, drivers, motorcyclists) (BMVI, 2018; GDV, 2017). In this study, safety-critical incidents refer to actions performed by the roadside agents of the behaviour-relevant changes who perform actions or behaviours that can potentially risk the safety of the driver or themselves. In the instances that we developed virtual experience, we make sure that it is difficult for an accident to happen, as the events take place at a safe distance from the driver; however, braking is mostly required from the driver.

All changes encountered by participants were triggered at the same geographical point along the path to ensure that all participants would experience the events at a similar time, depth of view, and perspective. The 72 changes were equally distributed in 36 pairs, divided into 12 pairs per complexity level (Appendix-Table 3). Every pair involved one behaviour change, either behaviour-relevant or behaviour-irrelevant, and one property change. The changes of each pair were triggered at the same point in the path, with the behaviour change always occurring first and the property change following (Fig. 4). This organisation of changes in pairs was designed to systematically study how the behaviour changes interfere with the detection of property changes that chronologically follow. We systematically manipulate the temporal proximity between the changes of each pair, by dividing the pairs into groups and assigning a time gap of 0, 1, 2, 4, 6, or 8 s between the changes. The selection of time gaps between the changes was based on previous studies on attentional blink, perceived duration, and event boundaries, suggesting that people need approximately 180–240 ms to detect visual stimuli and perceive duration (Jain et al., 2015; Efron, 1970), more than 500 ms to distinguish between stimulus (Sheppard et al., 2002; Raymond et al., 1992), 2–3 s to integrate an activity or even a few more seconds to encode meaningful events (the duration can be even longer depending on the nature task or other individual differences) (Zacks & Tversky, 2001; Swallow et al., 2018; Fairhall et al., 2014). As the literature provides different perspectives on the time gaps that affect perception, we combine the different perspectives and we define accordingly the test levels between a minimum at 0 s, and a maximum at 8 s. Therefore, this range of time gaps makes it possible to test the previous theories on the amount of time needed to register and detect high-level events.

The independent and dependent variables of the study are presented in Table 1. To facilitate the analysis of the pairs of changes we made sure that the time period between the pairs was at a minimum of 10 s, and more regularly between 10 s and 2 min (depending on the driving speed and behaviour of the driver, as well as the traffic lights).

### Apparatus

Simulated driving in the virtual environment is practically realised with a physical vehicle controller consisting of full steering and braking controls; the virtual driving environment and on-road interactions are presented through an HTC Vive headset equipped with an add-on eye-tracking device (by Pupil Labs). The VR headset provides a field of view of approximately 112$^{\circ }$ horizontally and 116$^{\circ }$ vertically, an image refresh rate of 60–90 Hz that reduces simulator sickness (Fig. 2) and presents a $1080\times 1200$ pixel image to the display of each eye, with partial stereo overlap. The headset makes use of 360$^{\circ }$ manoeuvring capability in conjunction with eye movement tracking at 120 Hz.

We recorded the gaze and driving behaviour of the participant, the detection performance, the egocentric view of the dynamic virtual scene, as well as a birds-eye view of the dynamic movement of the car in the urban environment (Table 1). Participants were seated in a car seat in order to maintain the same settings for all participants considering the position and distance of steering wheel and the mirrors. The setup allowed measurement of the participant’s speed, braking response, and steering angle while driving through the virtual environment, as well as the head movements (rotation and translation) based on the headset tracking. Two buttons located on the sides of the steering wheel were used for the responses of the change detection task. A speed limit of 30 km/h was enforced for the simulated vehicle to control the overall experience between participants and maintain consistency for the time proximity variable among the changes.

### Procedure

Before the test, participants were given a brief standardised explanation of the test protocol and completed a consent form according to the guidelines of the Swedish Ethical Review Authority. Participants were instructed to drive as they do everyday, respect traffic rules, and follow automated speech instructions from the GPS to their destination while they were also asked to perform the change detection task. Participants were informed about the distinction between the different types of changes through a video with examples, and they were then instructed to press one of two distinct buttons, on the right and left side of the steering wheel, to record a successful detection of a new change (using the right button to mark behaviour changes and the left to mark property changes). These two distinct buttons are used as a systematic approach to reassure that participants respond to the corresponding changes (even for changes with close time proximity) without interrupting the task with oral verification. The participants then proceeded to execute a familiarisation trial session of embodied driving in a VR driving simulator through a virtual test environment. Participants drove freely in the test environment to become accustomed to virtual driving and to practice some change detection trials. The test commenced when the participants reported they were comfortable driving in the virtual environment. Familiarisation sessions for each participant lasted approximately 5 min. Then, an eight-point calibration of the eye-tracking device was performed, and after that, participants started the test. Participants drove along a route following the instructions from the oral GPS, which guided them to the destination by providing information concerning the approaching turning points. The start, as well as the destination, was indicated with a sign and a verification by the GPS. Although there was no explicit time limit, participants completed the task in 20–30 min on average. A questionnaire followed the test session that included demographic questions concerning gender, age, driving experience, and gaming experience. The questionnaire also included an evaluation of motion sickness, perceived performance, and fatigue based on the NASA Task Load Index (TLX) (NASA, 1980). The entire study lasted approximately 45–50 min for each participant.

### Data coding and interpretation

Button presses (in response to a positive change detection) are counted as successful detections if conditions (C1–C2) are met: (C1) the participant pressed the *right-sided* button after a behaviour change, or the *left-sided* button after a property change; and (C2) either of the button presses occur before the next change occurs. To ensure the robustness of the findings, we exclude cases of misjudgement from participants using the concept of a *time window*, motivated by previous change detection studies (Levin et al., 2019; Berger & Kiefer, 2021). Here, the time window is the legitimate interval of time between the occurrence of a change and a Reaction Time (RT) cut-off time point determined based on the mean RT plus three standard deviations calculated across all subjects for all conditions. This way, the time window of 4.1 s is derived. Use of this time window implies that misses were accounted for when participants did not press the button in response to a change within the legitimate cut-off time window, or when participants press the button outside of the cut-off time window. To verify this approach, we compare the number of button presses *inside* and *outside* the legitimate response time window. We observe that 91.8% of the all button presses (both left, and right) occur within the legitimate time window of 4.1 s. The rest 8.2% of button presses occur outside of this time window, and they are not considered in our analysis. Based on this cut-off time window, we calculate that the overall detection performance was 55.9% across conditions.^2^

The afore-stated method of utilising a legitimate time interval is necessary since oral verification of the change detection was not requested, and the think-aloud method during driving was not encouraged in order to control for attentional distraction from the driving task, as per the experimental design described in similar studies (Rosenbloom & Perlman, 2016; Carney et al., 2018). Furthermore, we designed the placement of objects and agents in the scene in such a manner that no other aspect can be misjudged or misinterpreted as a change for the specific time point when the change occurs. These design decisions were made to keep the task as naturalistic as possible while at the same time to facilitate the analysis by making sure that participants’ responses correspond to actual changes.

## Results

All 80 participants completed the task in 45–50 min, with no extreme cases of very high or very low overall performance on the change detection task. The analysis of the questionnaires assessing self-reported task load showed that the 76.6% of participants rated the task as medium-to-high in mental demand. Moreover, 65.9% of participants were moderately confident in their responses concerning their performance. The dataset consisted of 45.5% frequent divers, 39.8% occasional drivers, and 14.7% new drivers. As the factor of driving experience was unbalanced, we did not include this analysis in the results.

### Strategies to mitigate visuospatial complexity

**Driving performance.** We assessed the driving performance per complexity level based on the average speed, the average time of completion, and the number of times drivers used the brakes. Because the task was interactive, the time of task completion is related to participants preferences and driving behaviour. Considering that the route distance for all complexity levels was equal, the analysis of the average time of completion per complexity level showed that participants spent on average 5.56 min to navigate the low complex environment, 5.57 min for the medium, and 6.87 min for the high complex scene (we exclude the time for navigation between transitional spaces among the complexity levels). ANOVA analysis showed a significant effect of visuospatial complexity on the average duration of crossing the scenes $\textit{F}(2,237) = 13.631, \textit{p} < .001, \eta _{p}^{2} = .103$. Post-hoc comparisons using the Tukey HSD test indicated that the mean time of completion for low and medium complexity level had no significant difference ($p = .963$), while the mean duration in high complexity level was significantly longer than in medium and low complexity ($p < .001$).

Moreover, the driving task took place in an urban environment where, by regulation, the speed limit was 30 km/h. As a result, there was not much diversity between the average driving speed recorded by participants among the different environments (26 km/h for low complex, 25 km/h for medium complex, and 18 km/h for high complex). Concerning the rate at which the brakes were used by participants, ANOVA analysis showed a significant effect of visuospatial complexity on the number of brake hits recorded (referring to the times a participant pressed the brake pedal along the route), with more brake hits recorded in the lower visuospatial complexity environment, $\textit{F}(2,237) = 30.161, \textit{p} < .001, \eta _{p}^{2} = .489$. On average 632 brake hits were recorded in the low complexity level, 536 in the medium, and 358 in the high complexity. Post-hoc comparisons using the Tukey HSD test indicated that the average number of brake hits in low complexity ($M = 632$, SD = 141) was significantly higher ($p = .009$) than in medium complexity ($M = 536$, SD = 104), and similarly in medium complexity the number of break hits was significantly higher ($p < .001$) than in high complexity ($M = 358$, SD = 106). These results suggest that an increase in visuospatial complexity leads to a decrease on the average number of brake hits recorded by participants.

**Fig. 5:**
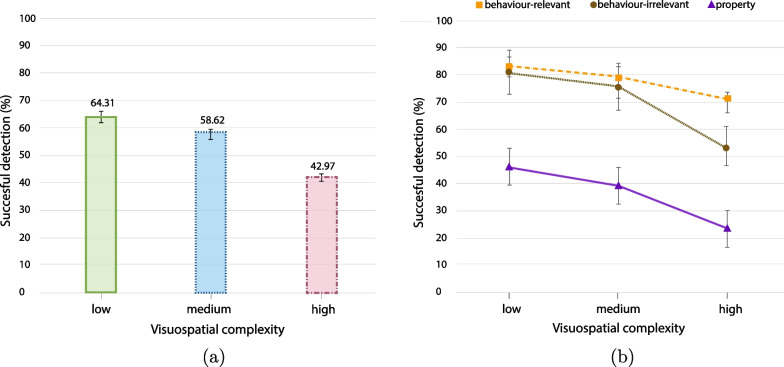
Analysis of detection rate among visuospatial complexity levels (all error bars are standard errors).** a** Overall detection rate per visuospatial complexity level,** b** detection rate among change types and complexity levels.

**Change detection.** We analysed the detection performance based on two types of button presses: left button for property changes, and right button for behaviour changes (both relevant and irrelevant). Participants on average missed 44.1% of the changes across all conditions. Participants detected fewer changes as the level of visuospatial complexity increased (Fig. 5a). Participants also detected overall fewer property changes than behaviour changes. This outcome was expected as property changes always follow a behaviour change and they are less relevant to the driving task (Fig. 5b). A $3 \times 3$ ANOVA between visuospatial complexity levels (low, medium, high) and change type (behaviour-relevant, behaviour-irrelevant, property) was conducted with the percentage of successful detections as the dependent variable. The results suggest a significant overall effect of visuospatial complexity, $\textit{F}(2,711) = 45.922, \textit{p} < .001, \eta _{p}^{2} = .114$, but not of change type, $\textit{F}(2,711) = 2.692, \textit{p} = .068, \eta _{p}^{2} = .008$. The interaction between the visuospatial complexity and the change types was also significant, $\textit{F}(4,711) = 4.443, \textit{p} = .001, \eta _{p}^{2} = .024$.

While the detection rate generally decreased as the level of visuospatial complexity increased, this reduction was mitigated for the behaviour-relevant changes in comparison to the other types of changes (Fig. 5b). Specifically, we calculated the rate of change for the detection performance as complexity increases.^3^ For the behaviour-relevant changes, the slope or the rate of change (ROC) between low and medium complexity level is $-3.9\%$, while between medium and high is $-9.02\%$. For the behaviour-irrelevant changes, ROC between low and medium is $-4.8\%$ and between medium and high is $-22.7\%$. Finally, for property changes the rate is $-7\%$ between low and medium, and $-15\%$ between medium and high. This analysis shows that the performance rate declines more radically for behaviour-irrelevant and property changes as complexity increases, especially between medium and high complexity levels (average decrease $-19.2\%$), while this rate appears smaller and more stable for the behaviour-relevant changes among all complexity levels (average decrease $-6.4\%$). Based on this observation, we performed paired t-tests focusing on the comparison of detection performance between behaviour-relevant and behaviour-irrelevant changes for low, medium and high complexity levels. The results suggest that the detection performance was significantly better for behaviour-relevant changes than for behaviour-irrelevant changes in high complexity environments, $\textit{t}(79) = 7.206, \textit{p} < .001, \textit{d} = .8$, while performance was very similar between these types of changes for the low and the medium complexity environments (low: $\textit{t}(79) = 1.299, \textit{p} = .198, \textit{d} = .14$, medium: $\textit{t}(79) = .366, \textit{p} = .715, \textit{d} = .04$). This outcome indicates that the detection rate was decreasing with a different rate between the medium and high complexity for these two types of changes, with behaviour-irrelevant changes recording a sharper decline.^4^

The overall analysis of driving behaviour and detection performance indicates that participants altered their driving and gaze behaviour as a result of a change in visuospatial complexity. Specifically, as the level of visuospatial complexity increased, participants moved slower, make fewer brake hits, and detected less changes especially for behaviour-irrelevant and property changes.

**Fig. 6:**
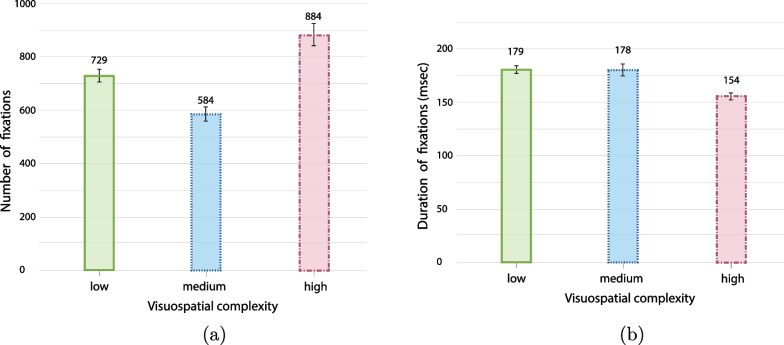
Analysis of fixations among visuospatial complexity levels (all error bars are standard errors).** a** Average number of overall fixations,** b** average duration of fixations

### Gaze behaviour adjustments

**Fig. 7:**
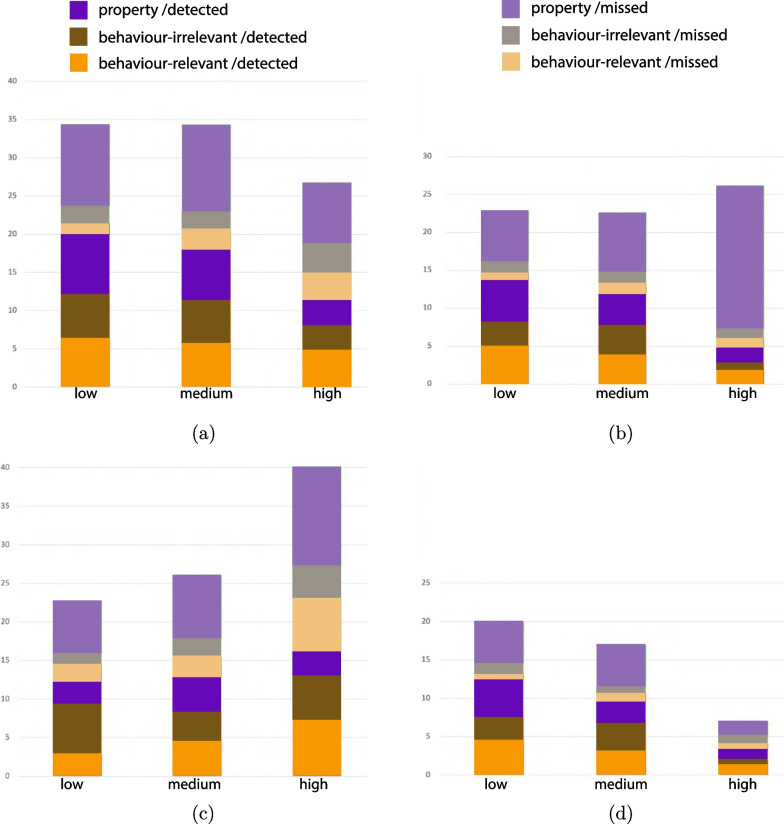
Detection of changes (%) in relation to the corresponding gaze behaviour (fixations on AOI of the change) for the change type and visuospatial complexity level. **a** Fixations on AOI only before the change, **b** fixations on AOI only after the change, **c** no fixations on AOI of the change, **d** fixations on AOI before & after the change

An overall analysis of gaze behaviour along the timeline of the changes, in combination with the change detection performance, indicates that gazing at an AOI of a target before and after a change is indicative for detection, but it does not necessarily lead to successful detection. Moreover, different gaze patterns are observed in the different change types and complexity levels before and after a change occurs (Fig. 7). Fixations were extracted based on guidelines of the eye-tracking device (Pupil Labs), with fixation duration between 100–500 ms and dispersion between $0.7^{\circ }$–$1.3^{\circ }$. Fixations on AOIs are defined as fixations landed in the 3D environment around the agent or the object (saccades over this AOI were excluded from this analysis).

**Analysis of fixations.** ANOVA analysis on the effect of visuospatial complexity on the number of fixations per minute showed a significant effect of visuospatial complexity on the number of fixations $\textit{F}(2,228) = 6.633, \textit{p} = .002, \eta _{p}^{2} = .56$, with the lowest average number recorded in the medium complexity environment ($M = 176$), while the highest average number of fixations per minutes was recorded in the low complexity environment ($M = 214$, high complexity environment recorded $M=191$). A post-hoc comparison using the Bonferroni test (Bonferroni, 1936) shows significant differences between low and medium complexity ($\textit{p} = .001$) but not between low and high ($\textit{p} = .091$), or medium and high ($\textit{p} = .456$). The average duration that participants spent in every complexity level was also affected by the visuospatial complexity level. As described in “Driving Performance” paragraph, participants spent significantly more time in high complexity than the other two environments (on average 5.56 min in the low, 5.57 min in the medium, and 6.87 min in the high complex environment). This outcome shows that participants were slower in the high complex environment, and they recorded a moderate number of fixations per minute. Nevertheless, the average amount of fixations recorded during the time they spent in the high complexity environment was higher than the other environments. Specifically, the analysis of the average number of fixations recorded showed the highest number of fixations in the high complexity environment (729 fixations in low, 584 in medium, and 884 in high, $\textit{F}(2,228) = 21.082, \textit{p} = .002, \eta _{p}^{2} < .001, \eta _{p}^{2} = .16$), suggesting that participants slowed down to be able to perform more overall fixations (Fig. 6a).

Concerning the average fixation duration in the three complexity levels, one-way ANOVA showed an overall significant effect of complexity on the average duration of fixations $\textit{F}(2,237) = 9.905, \textit{p} < .001, \eta _{p}^{2} = .083$ (Fig. 6b). Moreover, a post-hoc pairwise analysis showed a significant difference between low and high complexity levels $\textit{p} = .001$, as well as between the medium and high $\textit{p} < .001$, but not between low and medium $\textit{p} = .43$. These results indicate that with increasing complexity the overall number of fixations increased, while the average duration of fixations decreased.

**Table 2:**
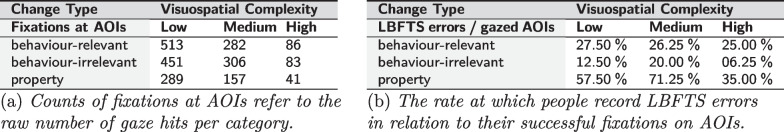
Gaze behaviour with respect to successful fixations at AOIs

**Looked-but-failed-to-see (LBFTS) errors.** Overall, 55.9% of all changes have been detected by participants based on the button presses metric. Nevertheless, 60.2% of all changes received direct fixation (for more than 100 ms) on the relevant AOIs. This result suggests that fixations on AOI do not correspond directly to successful detections, showing that saccades and peripheral vision can also contribute to successful detection (Table 2a, Fig. 7c). While at the same time gazing at the relevant AOI does not necessarily mean that participants detect the change (Fig. 7a, b, d). We further analyse the cases where we record fixation on the relevant AOI but no button presses were recorded. These cases were classified as LBFTS errors based on two criteria: “did the participant look at the AOI of a change before *and* after the change happened?” (yes or no), and “did the participant respond to the change?” (yes or no). A case was classified as an LBFTS error when participants failed to detect a change despite having gazed at the corresponding AOI for more than 100 ms during both the time window of 4 s before and the time window 4 s after the trigger time of a change (White & Caird, 2010). In this time window, all targets were visible to the participant for all the trials. Further analysis with a time window of 2 s before and 4 s after the change did not substantially alter the results.^5^

**Fig. 8:**
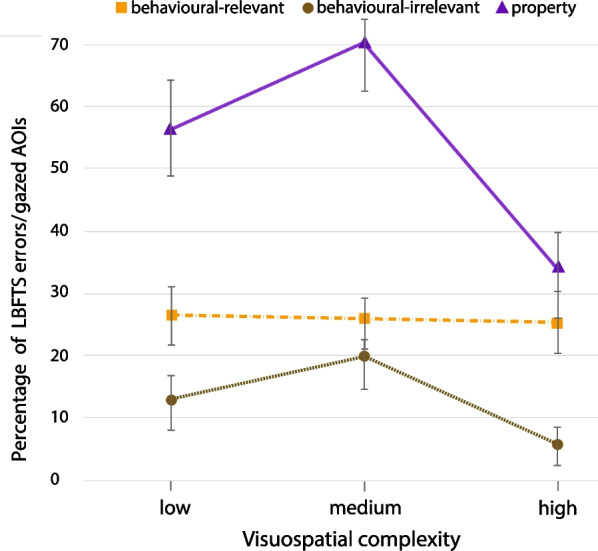
Average number of LBFTS errors for number of successful gaze on AOI, analysed based on change types and visuospatial complexity levels (all error bars are standard errors)

On average, participants recorded 31.2% LBFTS in all conditions, however the property changes recorded fewer changes than the rest of change types and higher visuospatial complexity also recorded reduced rate of LBFTS errors (Table 2b). A 2-way visuospatial complexity (low, medium, high) x change type (behaviour-relevant, behaviour-irrelevant, property) ANOVA with the rate of LBFTS errors as the dependant measure showed significant difference across the three visuospatial complexity levels, $\textit{F}(2,711) = 4.768, \textit{p} < .001, \eta _{p}^{2} = .013$, and the change types $\textit{F}(2,711) = 31.281, \textit{p} < .001, \eta _{p}^{2} = .081$. The behaviour-irrelevant and property changes produced similar patterns of LBFTS errors across visuospatial complexity levels, while the rate of LBFTS errors for behaviour-relevant changes did not vary significantly across complexity levels (one-way ANOVA: $\textit{F}(2,239) = 0.12, \textit{p} = .988$; Fig. 8).

**Fig. 9:**
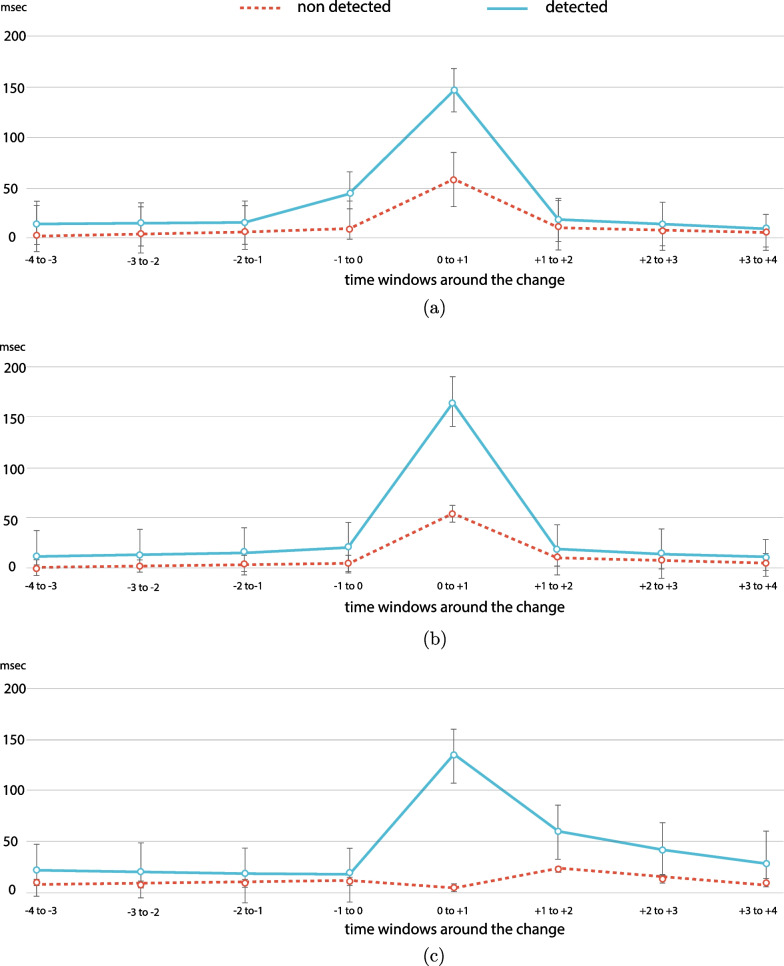
Average number of ms with fixations on AOI along the timeline of a change. The time window analysed is 4 s before to 4 s after a change. We report on fixations in cases of detected and non-detected changes for all change types (all error bars are standard errors).** a** Fixations on AOIs of behaviour-relevant changes before and after the change,** b** fixations on AOIs of behaviour-irrelevant changes before and after the change,** c** fixations on AOIs of property changes before and after the change.

**Fig. 10:**
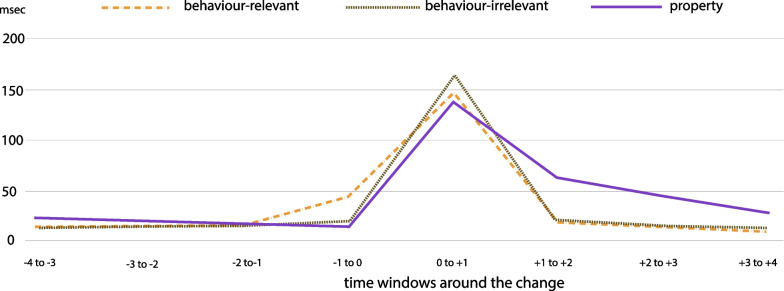
Fixations on AOI for detected changes in relation to time window close to the change and the change type

**Attentional lingering and monitoring attention.** Analysis of fixations on AOI along the time window of 4 s before a change until 4 s after a change show that gaze patterns effectively differentiate *detected* versus *missed* changes (Fig. 9). The gaze analysis also suggested that participants developed different gaze patterns for different types of changes. ANOVA analysis between 8-time windows (4 before and 4 after the change) x 3 (change types) x 2 (detected or missed) showed that participants gazed at the AOIs of detected changes significantly longer ($M = 30.9$ ms) than the AOIs of missed changes ($M = 25.$4 ms); $F(1,2844) = 8.87; \textit{p} = .003, \eta _{p}^{2} = .003$). Moreover, there were more fixations during the first second after the change for detected changes relative to missed changes. ANOVA analysis also confirms that fixations on AOIs differ significantly along the timeline of the change ($F(7,2844) = 209.8, \textit{p} < .001, \eta _{p}^{2} = .269$). These results show that gaze behaviour is likely to influence the detection performance and that changes or events that involve other humans attract attention independently of the outcome of the detection task (Fig. 10).

**Fig. 11:**
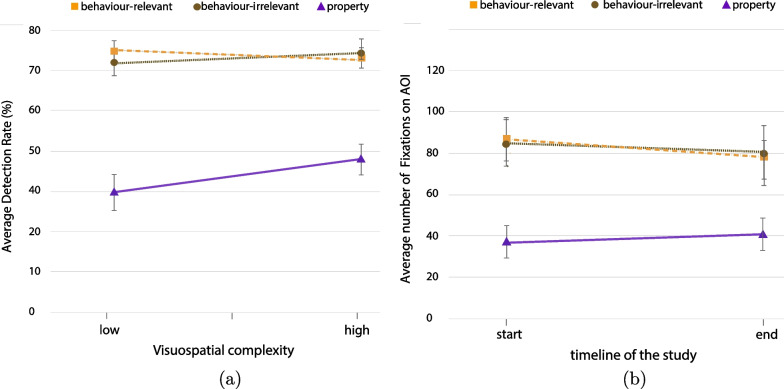
Analysis of learning effect per type of change calculated based on two metrics (all error bars are standard errors).** a** Learning effect per type of change calculated based on fixations on AOIs,** b** learning effect per type of change calculated based on detection rate.

**Fig. 12:**
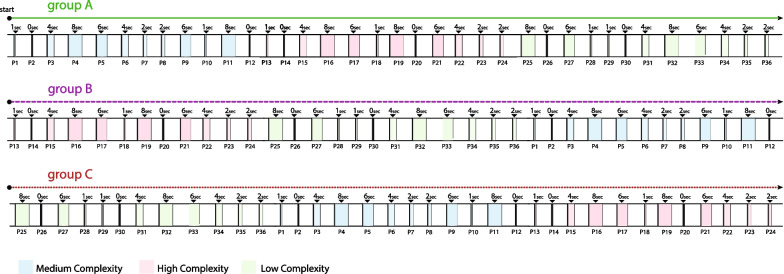
Structure of the experimental session for the three groups of participants

A closer comparison of change types for the time window just before the change, specifically the time window $-1$ to 0 s, indicates that participants employed pre-change monitoring attention for behaviour-relevant changes which was not the case for the other change types (Fig. 10). Moreover, ANOVA analysis showed a significantly greater number of fixations on AOIs for behaviour-relevant changes ($M = 26.344$ ms) than for behaviour-irrelevant changes ($M = 12.2$ ms) independently of the detection or miss of the change, $\textit{F}(1,316) = 22.353, \textit{p} < .001, \eta _{p}^{2} = .066$. These results suggest that many times people gaze at the AOI of a change before the change occurs. This behaviour known as “anticipatory gaze” was exhibited in cases where (vulnerable) street users were involved and that participants judged as safety-critical and they differ significantly from the rest of safe interactions with street users (Fig. 9a, b). Additionally, we observed a lingering effect for property changes, where participants produced increased gazing at AOIs even 3 s after the change; that was not the case for behaviour changes (Fig. 9c). We hypothesise that this effect might have been caused by the fact that behaviour changes were often followed quickly by a property change, so participants learned to move on after a behaviour change (see organisation of changes in Appendix-Fig. 12). Analysis of the learning effect based on two metrics—detection rate and fixations on AOIs—confirms an effect only for property changes and only for the metric of detection rate (Fig. 11). Specifically, the comparison between the first and the last session of the study shows no significant difference in detection rate, $F(1, 3057) = 4.474, p = .034, \eta _{p}^{2} = .001$, or in the number of fixations on AOIs, $F(1, 4436) = .969, p = .325, \eta _{p}^{2} = .000$. However, the analysis of property changes separately shows a significant learning effect in the metric of detection rate, $F(1, 2236) = 10.861, p = .001, \eta _{p}^{2} = .005$, but not for fixations on AOIs $F(1, 2236) = .975, p = .325, \eta _{p}^{2} = .000$.

**Temporal load and detection performance.** As a reminder, the change detection task was organised based on pairs of changes, where the participant first encountered a behaviour change followed by a property change (Fig. 4). We examined the detection rate and the RT for the successfully detected property changes in relation to the time proximity from the behaviour changes. Here, time proximity serves as an independent variable (predictor) with six levels, where the time gap between the changes is one of 0, 1, 2, 4, 6, 8 s. A one-way between-participants ANOVA reveals a significant effect of time proximity on the change detection for all levels, $\textit{F}(5,474) = 14.286, \textit{p} < .001$, with participants performing significantly worse when two changes happened simultaneously. Post-hoc comparisons using the Bonferroni test show that the detection score for the 0 s level ($M = 24.8\%$, SD = 21.3%) was significantly lower than the rest of the levels (e.g. the level 2 s recorded $M = 43.8\%$, SD = 27.6%, or 6 s with $M = 38.5\%$, SD = 24.3%). Between the rest of the groups (1–8 s), no significant difference was found with respect to the detection rate (ANOVA: $\textit{F}(4,399) = 1.72, \textit{p} = .163$) (details in “Appendix C”).

## Discussion: results and applied implications

The study examines attentional performance in the context of everyday driving under diverse, systematically manipulated visuospatial complexity conditions. The key direct results of the study reveal two key findings pertaining to attentional compensation as a result of visuospatial complexity changes, and adaptation of gaze behaviour patterns in relation to the change types encountered in the task. Furthermore, these basic results are also interpretable from an application viewpoint given their implications in settings such as driving education and development of driving skill-testing methods and human-factors guided development of AI-based driving assistance systems.

## Key behavioural results

**Result A: Attentional compensation in response to visuospatial complexity**

An increase in visuospatial complexity of the perceived stimuli (i.e. the urban driving environment) affects gaze behaviour, the driving behaviour, as well as change detection performance. High visuospatial complexity environments result in more fixations with shorter duration, indicating a more exploratory gaze behaviour, while lower detection rate of changes accrue when the complexity increases. Moreover, the driving performance is affected too, with lower speed and less frequently braking in high complexity environments, possibly to provide the necessary time for the driver to explore the scene with more fixations. These results are in line with previous studies (e.g. by Hulleman et al. (2020); Beanland et al. (2017)), suggesting that an increase in complexity can increase the difficulty of an attentional task, thereby leading to changes in behaviour, and limitations in attentional performance.

Specifically for driving, even though drivers’ performance and change detection performance is generally worse in urban than rural environments (Beck and Levin, 2003; Beck & Trafton, 2007; Wright et al., 2000), some conflicted results have also been recorded, suggesting that better performance in urban environments is a result of legible layouts and signalling that assist in the change expectations (Koustanaä et al., 2012). In this study we analyse the streetscape further than the semantic characterisation of urban, suburban and rural, providing a comprehensive and systematic exploration of visual, spatial and interactive aspects of the dynamic driving environment. The outcome shows that while an incremental increase in the level of visuospatial complexity leads to a decline in change detection performance, the relevance of the changes to the driving task is also significant. In particular, participants are more successful at detecting changes that involve other road users, especially when the road users are part of a potentially hazardous event or are interrupting the driving experience (e.g. by crossing the street, overtaking, or riding a bike in front of the driver). The gaze behaviour analysis also suggests that participants gaze at other road users closely before and after the changes independent of the detection performance. These results confirm previous research suggesting that people can better detect targets related to the current activity (e.g. for driving this can be traffic lights) as well as targets plausible to change (e.g. cars, motorcyclists, pedestrians) rather than other more nominally stable objects (e.g. signs, trees) in the scene (Beanland et al., 2017; Lee et al., 2007; Beck et al., 2004). The results also suggest that participants are able to distinguish between elaborate and detailed variations in the behaviour of other road users and prioritise their attention accordingly, resulting in better performance in changes involving road users in safety-critical situations (e.g. behaviour-relevant changes are better detected than behaviour-irrelevant changes even if both types of changes involve road users).

Moreover, the visuospatial complexity of the environment affects participants’ gaze behaviour with respect to the number and duration of fixations, the fixations on relevant AOIs, as well as the rate of LBFTS errors. Participants’ deployment of attentional strategies to mitigate the effects of complexity is dependent on the change type: anticipatory gaze behaviour is crucial for behaviour-relevant changes but not for property changes and behaviour-irrelevant changes. On the contrary, mitigation strategies for the effects of high complexity on LBFTS errors are effectively deployed for property changes and behaviour-irrelevant changes, but behaviour-relevant changes are not affected.

Specifically, the analysis of fixations shows that gaze is more exploratory as complexity increases resulting in more overall fixations, shorter durations, and less fixations finding their target (as denoted by fixations on AOIs). This observation shows that detecting a relevant target is harder when visuospatial complexity is high. Moreover, the rate of LBFTS errors reduces in high complexity for behaviour-irrelevant and property changes, suggesting that gaze behaviour was more efficient when complexity increased, as participants succeeded in detecting the changes that they gazed at (even though the average fixations on AOIs are overall lower in high complexity environments). Nevertheless, LBFTS errors for behaviour-relevant changes are not affected by the visuospatial complexity levels—a stable LBFTS error rate is recorded for all levels of complexity (approximately 26%)—indicating that behaviour-relevant changes attract participant’s attention in a more consistent way, even if the change is not detected, as they involve other road users and they are more relevant to the driving task.

**Result B: Anticipatory gaze and attentional disengagement**

A detailed gaze analysis along the time window of the changes shows that behaviour changes attract attention even if the change is not detected, which is not the case for property changes. These results are in line with our previous observation concerning differences in gaze patterns with respect to fixations on AOIs and LBFTS errors among the change types. Specifically, gaze behaviour analysis pertaining to behaviour-relevant changes shows that anticipatory gaze is crucial for detection. A significant increase in gaze hits on AOI is recorded 1 or 2 s before a behaviour change, which predicts successful detection. However, independent of whether successful detections are triggered, participants exhibit a significant number of fixations on behaviour changes. This gaze behaviour is considered monitoring attention of drivers towards other road users in order to assess potential hazards. In comparison, property changes do not result in a significant increase of fixations on AOIs in cases where changes are not detected. For property changes, it is clear that allocating attention to AOIs happens mostly during the second after the change occurs and only when the change is detected. Further analysis, both quantitative and qualitative, of participants’ gaze behaviour in relation to fine-grained differences in interactions patterns and analysis of effects of event duration on attention are themes that need to be addressed in future studies.^6^

Analyses of anticipatory gaze and attentional disengagement demonstrate the ability of participants to evaluate situational hazards and allocate attention accordingly, with behaviour-irrelevant and property changes not receiving anticipatory or monitoring attention before the changes as the behaviour-relevant changes do. Our expectation was to register more LBFTS errors for changes irrelevant to the driving task—both behaviour-irrelevant and property changes—in comparison with behaviour-relevant changes. However, LBFTS errors were also observed for behaviour-relevant changes. This observation, we believe, is related to a delay in gazing at the AOIs, resulting in not considering the event as a change but processing and reacting to it by braking or stopping the car and interacting with the agent. Therefore, in this case, LBFTS errors might be related more to delays in detection rather than to misses in information processing.

Examining the results of this study from the viewpoint of *perceptual load theory* (Lavie & De Fockert, 2003), we suggest that high visuospatial complexity increases the difficulty of a complex everyday task of detecting critical events while driving. In line with previous work on human perceptual and sensory limitations (Benoni & Tsal, 2012), and the link between perceptual load and suppressed neural circuits (Fougnie et al., 2005), this study demonstrates that people perform worse in change detection tasks when the visuospatial complexity is high, even for the types of changes that people otherwise can easily perceive (e.g. comparing the same set of salient targets in low complexity environments). In this study, the performance of participants was primarily affected by visuospatial complexity in the case of high complexity environments. Under these highly demanding circumstances, we observe attentional prioritisation by participants towards selective types of changes that were more relevant to the primary task of driving safely in the immersive environment. This behaviour by the participants did not vastly improve the overall performance but it did limit the effect of distractors, leading to better gaze control and detection performance for the subset of targets that involved road users in safety-critical situations. In line with previous studies (Murphy et al., 2016), these results suggest that high environmental complexity could drain surplus perceptual resources and thus reduce task-irrelevant interference leading to relatively better performance of the main task but compromised performance for secondary targets.

## Applied implications

**Driver education and testing.** The results of this work can lead to the development of novel testing and training techniques for drivers, e.g. through the provision of metrics for effective driver attention and attentional strategies deployed. Such metrics can serve as extensions of existing tests such as Drive Aware Task (DAT) (Feng et al., 2015) and Attention-Related Driving Errors Scale (ARDES) (Ledesma et al., 2015) under diverse systematically controlled environmental conditions, involving complex environmental structures and/or complex interaction events, as utilised in our research. As shown in this study, environmental and temporal complexity affect human performance on high-level visual processes such as change detection during driving, however, even in highly complex situations people are able to adjust their behaviour and prioritise attention towards safety-critical situations. This suggests that professional drivers especially can be trained and tested on how efficiently they decelerate in highly complex situations and challenging conditions, adjust their fixations to detect targets in diverse areas of interest (AOI), make attentional choices between events when necessary, and disengage from targets and distractors. For example, testing abilities and behavioural traits of professional drivers should include not only visual acuity tests but also cognitive tasks such as monitoring and assessing changes in the behaviour of other vulnerable road users (e.g. kids, older adults), anticipating crossing behaviours in busy urban areas, responding to occlusion events, and keeping high situation awareness with overhead checks in highly dynamic urban environments, monitoring blind spots, etc. Overall, investigating the impact of environmental complexity and temporal proximity among events based on systematic behavioural studies on driving, we suggest that metrics of driver’s skills should include strategic preservation of attention to highly relevant events and drivers’ education should involve training of attentional strategies based on the conscious knowledge of human’s physiological attentional limitations.

Considering attentional strategies in relation to environmental and temporal complexity, we posit that external characteristics (e.g. environmental, temporal) are not always enough to holistically explain drivers’ performance, as personal characteristics also play a critical role. Aspects of working memory capacity, attentional breadth, visual stability, spatial representation and spatial capabilities, preference in attentional strategies, etc., can be dependent on individual differences deriving from age, gender, culture, or other cognitive specificities (Andermane et al., 2019; Angelone & Severino, 2010; McPhee et al., 2004). Consequently, the evaluation and education of drivers should incorporate gaze and driving performance analysis taking into consideration the fact that different individuals may use different methods to address the same situation based on their skills. Moreover, novel educational techniques that involve knowledge of individual differences and focus on automatically identifying attentional failures related to attentional lingering, or excessive LBFTS errors during driving, can serve as an instrument for driving self-assessment in an educational context (e.g. self-assessment driving test in VR).

**Human-centred visual intelligent systems.** The behavioural outcomes of naturalistic behavioural studies on high-level human processes as the ones discussed in this work, can constitute the basis for precedent-based modelling of human everyday interactions between each other and with the environment. Recent work in human-centred AI focuses on incorporating knowledge of human behaviour, human abilities and preferences in artificial visual intelligence systems (Bhatt & Suchan, 2023, 2020), which can be valuable for anticipating and explaining human behaviour and interactions in domains such as autonomous vehicles and driving assistance systems (Suchan et al., 2021).

Specifically, human-centred explainable visual sense-making refers to the process of providing explanations for events and interactions between humans and the surrounding environment by analysing environmental features (e.g. clutter, motion, scene structure) and human behavioural patterns (e.g. head movement, body posture, speed and direction of movement). This process necessitates both high-level semantics and low-level visual computing, using a range of techniques developed in AI, Machine Learning, and Computer Vision. For the high-level semantics, human-centred representation and relational abstractions are supported by modelling of space, events, actions, motion, and (inter)action, and they need to be grounded in real-world data of human experience. Overall, processing and semantic interpretation of large volumes of highly dynamic visuospatial imagery are central and psychology and behavioural research where data-centred analytical methods are gaining momentum.

## Summary

By replicating a real-world driving experience in the virtual environment, we use a change detection task to assess the attentional cost of visuospatial complexity as well as the adoption of mitigation strategies deployed by the drivers. This study demonstrates that visuospatial complexity of the environment and the type of perceptual targets involved have a direct influence on change detection. Overall, while visuospatial complexity affects gaze behaviour and detection performance negatively, the effects are mitigated for changes involving road users in safety-critical situations. Moreover, gaze behaviour analysis shows successful anticipatory gaze and quick attention disengagement from behaviour changes, thereby indicating efficient attentional strategies deployed by the drivers, especially in high complexity scenes. These results add to our understanding of precise circumstances under which people adapt their attentional strategies to compensate for an increase in task difficulty caused by external factors.

## Outlook

This research explores the landscape of naturalistic behavioural studies and the systematic investigation of fundamental questions in high-level visual processing, such as change blindness in the everyday embodied context of driving. Real-world or “in-the-wild” studies on driving behaviour have face validity and the distinct advantage of focusing on driving in its most naturalistic context. Nevertheless, this absolute naturalism can lead to problems with confounds, noise and other restrictions in the data. Virtual reality (VR) studies provide an alternative to real-world embodied empirical studies of human behaviour, in a controlled environment, with random assignment of participants, balanced experimental manipulations of conditions, and reliable datasets with physiological measurements such as eye movement data. Although in this VR study we develop a systematic way of testing high-level visual processing during driving and collecting multimodal behavioural data, faithfully replicating VR-based empirical studies in the real-world will be necessary to establish the correspondence between the methodologies needed for responsible generalisation of the outcomes, and this process constitutes a theme of emerging interest in our research.

Specifically, our research develops between behavioural studies “in-the-wild” and in VR to address questions on everyday complex cognitive tasks such as change detection, active navigation (Kondyli & Bhatt, 2021, 2018; Kondyli et al. 2018), event perception (Nair et al., 2022), etc. Of great importance in this work are the multimodal interactions between humans, as well as between humans and their surrounding environment, specifically, the effect of environmental features such as manifest cues, structural characteristics, etc. We establish a methodology to systematically explore the effect of environmental features and their combinations on embodied everyday behaviour such as in driving and navigation (Kondyli et al., 2020, 2021), and so, future work will expand towards other high-level cognitive processes including visual foraging, visual search, attentional priming (Kristjánsson & Kristjánsson, 2019), as well as towards the investigation of individual differences with respect to age, experience, gender, spatial cognitive skills, etc. (Andermane et al., 2019; Mian & Jaffry, 2020).

## Figures and Tables

**Table 3:**
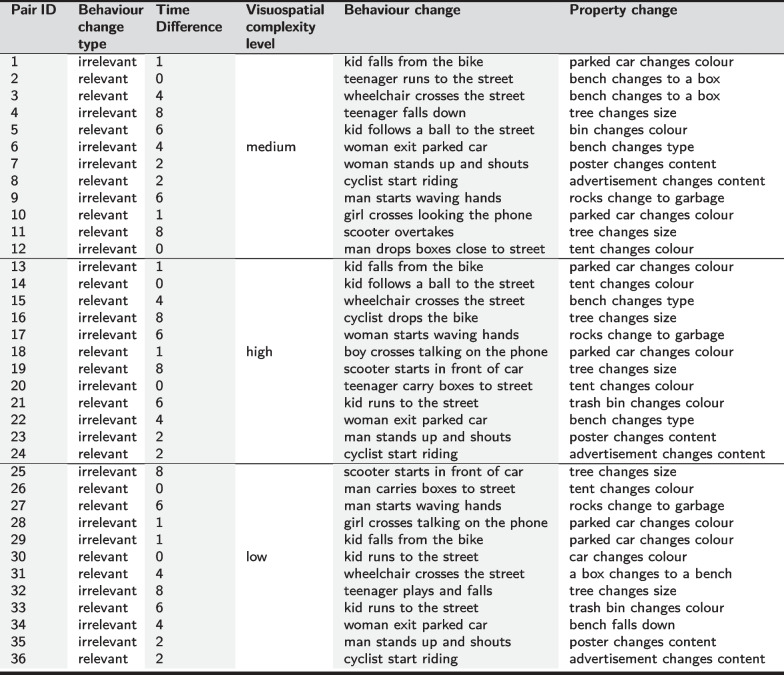
The structure of the 36 pairs of changes involved in the change detection task

**Table 4:**
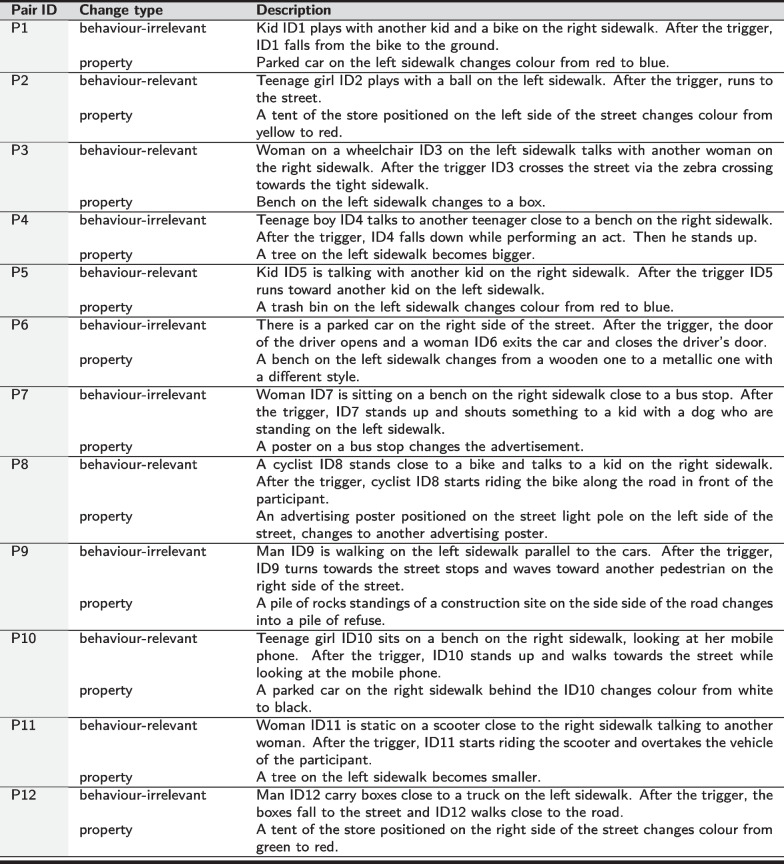
Event scenarios—part 1—medium visuospatial complexity

**Table 5:**
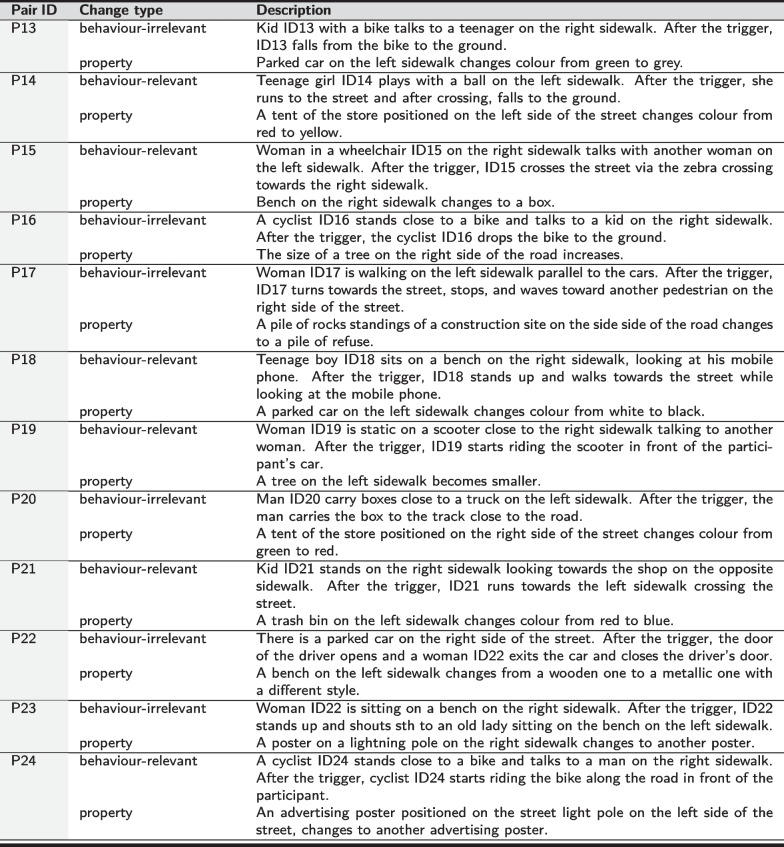
Event scenarios—part 2—high visuospatial complexity

**Table 6:**
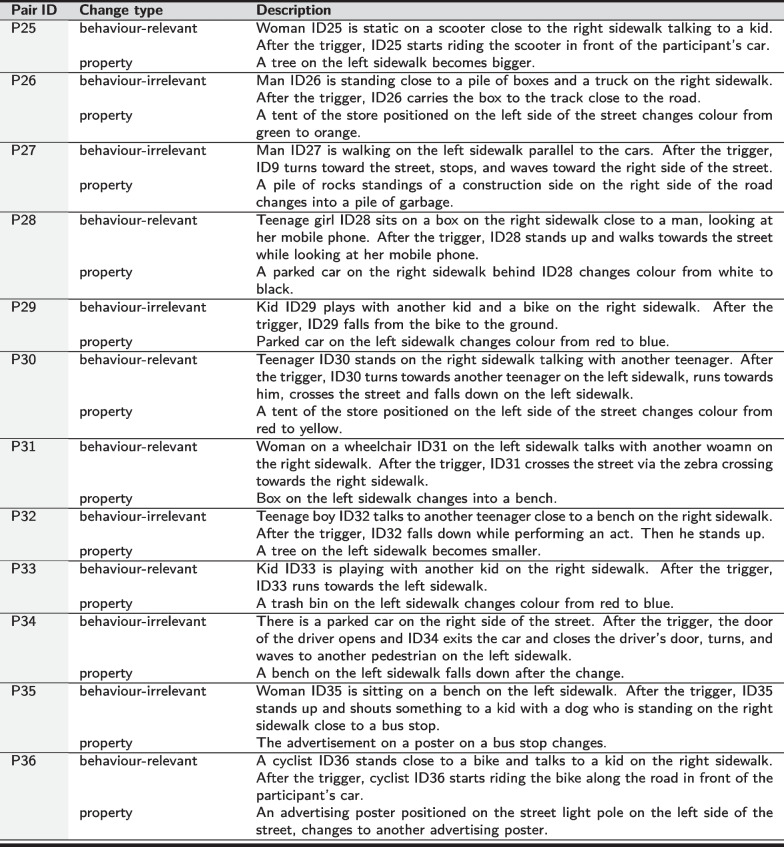
Event scenarios—part 3—low visuospatial complexity

**Fig. 13:**
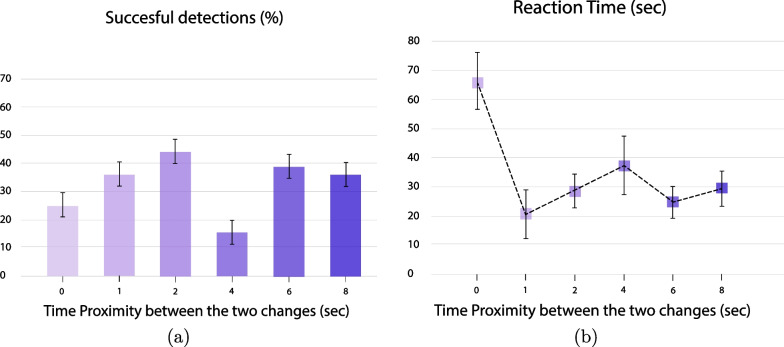
Detection rate and reaction time (RT) for detection of property changes (2nd change), with respect to the time proximity from the 1st change. All error bars are standard errors.** a** Detection rate for all groups of time proximity,** b** reaction time for all groups of time proximity

## Data Availability

During the review stage of this paper, anonymised data and all material related to the experiment will be made upon request through a secure/password protected URL by contacting the first author. Upon possible acceptance and final publication, all data can be made available publicly in conformance with the guidelines stipulated by the Swedish Ethical Review Authority (Etikprövningsmyndigheten).

## Appendix

### A. Visuospatial complexity model

In this study, we use a cognitive model of visuospatial complexity developed in our previous work—presented in Kondyli et al. (2020) and Kondyli et al. (2021)—to investigate how the combination of visual and spatial attributes in real-world scenes affect driving. The model incorporates: quantitative (e.g. feature congestion, subband entropy, luminance, street size), structural (e.g. repetition, symmetry), and dynamic attributes (e.g. direction of motion, number of moving objects, speed) extracted from systematic analysis of real-world scenes. These are combined with high-level characterisation of complexity based on qualitative evaluation of complexity and behavioural data. Based on this model, we developed a range of driving environments in VR that correspond to different levels of visuospatial complexity. We manipulated:

- the number of objects in the scene per metre, the objects’ colours and size, the size of the street, and the clutter in the frames;
- the level of symmetry in the structure of the scene, as well as the repetition and order of buildings, trees, and other street objects; and
- the number of vehicles, pedestrians, and cyclists, and their facing direction, direction of movement, and speed.

Example scenes from the developed environments are demonstrated in Fig. 3.

### B. Participants’ groups

To mitigate the effect of learning among the types of changes that appear with small differences in all three complexity levels, we employ three possible sequences of encountering the visuospatial complexity levels. Participants were randomly assigned to one of the three groups A, B, and C and experienced the levels of complexity in a different order (Fig. 12). The time between the pairs of changes was regulated in order for the pairs to be distinctive from each other and easier to analyse. The minimum time was 10 s, but more regularly it was between 10 s to 2 min, depending also on the driving speed and behaviour of the driver, as well as the traffic lights.

The structure of the 36 pairs of changes along the driving route of the study and the characterisation of the pairs based on the temporal proximity, change type, and visuospatial complexity level are described in Table 3. The description of every scenario involved in these 36 pairs of changes is presented in detail in Tables 4, 5, 6.

### C. Analysis of temporal proximity

These results suggested that time proximity can be considered an additional factor of task difficulty, while the effect was mostly absorbed by the detection for property changes, while the detection performance of behaviour changes mostly remained intact.

**Analysis of Detection Rate.** The analysis showed that the probability of detecting a change varies among the time proximity levels, as illustrated in Fig. 13a. We consider the time proximity level 4 s an outlier, as a closer analysis suggested that the extensive duration of the behaviour change in the particular scenarios involved in this group was the major factor for the low detection performance recorded. A one-way between subjects ANOVA revealed a significant effect of time proximity on the detection rate for all levels $\textit{F}(5,474) = 14.286, \textit{p} < .001$. Post-hoc comparisons using Bonferroni test indicated that the detection score for the 0 s level ($M = 24.8\%$, SD = 21.3%) was significantly lower than the rest of the levels (e.g. with level 2 s recorded $M = 43.8\%$, SD = 27.6%, or 6 s with $M = 38.5\%$, SD = 24.3%) (the case of 4 s is consider an outlier and is exclude from this analysis, $M = 15.2\%$, SD = 21%). No significant differences were found between the levels 1–8 s ($\textit{p} > 0.56$).

Especially the group with time proximity 0 s, recorded the lowest performance for property changes and the higher behaviour changes. The results suggest that people prioritise behaviour changes over property changes particularly when the time proximity in very short (0 or 1 s). A *t*-test analysis between the behaviour and the property changes showed significant difference of the means across the time proximity groups ($\textit{t}(938) = 18.047, \textit{p} < .001, \textit{d} = 1.16$). Moreover, ANOVA analysis showed that the effect of time proximity to detection performance is strongly significant for property changes ($\textit{F}(5,478) = 14.382, \textit{p} < .001$), but only marginally significant for behaviour changes ($\textit{F}(5,478) = 3.368, \textit{p} = .005$). These results suggested that time proximity can be considered an additional factor of task difficulty. The effect was mostly absorbed by the detection for property changes, while the detection performance of behaviour changes mostly remained intact.

**Analysis of Reaction Time (RT).** One-way ANOVA of RTs for the subset of changes that were detected (on average 36.6% of all property changes), showed a significant effect of time proximity on RT, $\textit{F}(5,926) = 4.440, \textit{p} = .001, d=$. Participants were significantly slower at detecting a property change that happened at the same time as a behaviour change (0 s level, $M = 1.63$, SE = 0.91), than in the case where there was a time gap of 1 to 8 s between the two changes (Fig. 13b). Post-hoc comparisons using Bonferroni test indicated that the mean score for the 0 s time proximity level was significantly different from the rest of levels ($\textit{p} < .001$). No significant difference was found between the rest of the levels (1–8 s).

Overall, the number of data points for each condition was not enough to provide a conclusive result on the effect of time proximity among all conditions; they did, however, indicate a significant difference between the changes which happen simultaneously and the ones with a few seconds time gap in between the pair. The results of detection performance, as well as the RT, suggest that detection performance for the second changes was significantly compromised when this change was performed simultaneously (time proximity of 0 s) to a behaviour change, with a probability of 24.8% to be detected, and an average RT of 1.633 s. For the rest of the groups of time proximity, a recovering trend was observed, with the best performance recorded in the condition of 2 s time proximity (with a probability of 43.8% to detect the change in an average of 1.306 s).

## Abbreviations

RT: Reaction time
VR: Virtual reality
AOI: Area of interest
LBFTS: Looked-but-failed-to-see

## Footnotes

1 Changes in objects that could be related to the driving task were not considered in this category, as for example the traffic light turned from green to red.

2 The total legitimate button presses correspond to 91.8% of all button presses; here, there may even be multiple presses for each detected change. To calculate the detection performance and the reaction time for each change per participant, we only consider the first (legitimate) button press recorded for every change. The number of such first-recorded instances of legitimate button presses corresponds to 55.9% of the total (legitimate) button presses across all conditions.

3 The rate of change (ROC) is calculated and presented as a percentage based on the formula ROC=previous value/current value.

4 Detection performance between behaviour and property changes was not further analysed because these are not directly comparable cases as a result of the experimental design.

5 For a window of 2s before and 4s after the change we observed a small increase in the average of recorded LBFTS errors to the rate of 0.3–1% overall, and specifically a 4.53% error rate for the behaviour-relevant changes, 2.76% for behaviour-irrelevant changes, and 13.24% for property changes. Consequently, no statistical difference between the recorded results between the two-time windows was observed.

6 Studies of interaction patterns could include utilising varying design conditions pertaining to position, gaze, gesture and orientation of interaction agents (e.g. vulnerable road users, pedestrians). Likewise, studies on event durations could include scenarios that evolve in different speed and include a different number of fine-grained activities. These positioned future studies merit a detailed treatment on their own, and hence are considered out of the scope of this paper.
